# A New Strategy Based on Smrho Protein Loaded Chitosan Nanoparticles as a Candidate Oral Vaccine against Schistosomiasis

**DOI:** 10.1371/journal.pntd.0001894

**Published:** 2012-11-29

**Authors:** Carolina R. Oliveira, Cíntia M. F. Rezende, Marina R. Silva, Ana Paula Pêgo, Olga Borges, Alfredo M. Goes

**Affiliations:** 1 Departamento de Bioquímica e Imunologia, Instituto de Ciências Biológicas, Universidade Federal de Minas Gerais, Belo Horizonte, Brazil; 2 INEB—Instituto de Engenharia Biomédica, Universidade do Porto, Porto, Portugal; 3 Instituto de Ciências Biomédicas Abel Salazar (ICBAS), Universidade do Porto, Porto, Portugal; 4 Faculdade de Engenharia da Universidade do Porto (FEUP), Universidade do Porto, Porto, Portugal; 5 CNC, Center for Neuroscience and Cell Biology; University of Coimbra; Coimbra, Portugal; 6 Faculty of Pharmacy, Pólo das Ciências da Saúde; University of Coimbra, Coimbra, Portugal; McGill University, Canada

## Abstract

**Background:**

Schistosomiasis is one of the most important neglected tropical diseases and an effective control is unlikely in the absence of improved sanitation and vaccination. A new approach of oral vaccination with alginate coated chitosan nanoparticles appears interesting because their great stability and the ease of target accessibility, besides of chitosan and alginate immunostimulatory properties. Here we propose a candidate vaccine based on the combination of chitosan-based nanoparticles containing the antigen SmRho and coated with sodium alginate.

**Methods and Findings:**

Our results showed an efficient performance of protein loading of nanoparticles before and after coating with alginate. Characterization of the resulting nanoparticles reported a size around 430 nm and a negative zeta potential. *In vitro* release studies of protein showed great stability of coated nanoparticles in simulated gastric fluid (SGF) and simulated intestinal fluid (SIF). Further *in vivo* studies was performed with different formulations of chitosan nanoparticles and it showed that oral immunization was not able to induce high levels of antibodies, otherwise intramuscular immunization induced high levels of both subtypes IgG1 and IgG2a SmRho specific antibodies. Mice immunized with nanoparticles associated to CpG showed significant modulation of granuloma reaction. Mice from all groups immunized orally with nanoparticles presented significant levels of protection against infection challenge with *S. mansoni* worms, suggesting an important role of chitosan in inducing a protective immune response. Finally, mice immunized with nanoparticles associated with the antigen SmRho plus CpG had 38% of the granuloma area reduced and also presented 48% of protection against of *S. mansoni* infection.

**Conclusions:**

Taken together, this results support this new strategy as an efficient delivery system and a potential vaccine against schistosomiasis.

## Author Summary

Schistosomiasis is one of the most important neglected tropical diseases and an effective control is unlikely in the absence of improved sanitation and vaccine. The selection of a suitable delivery system and an adjuvant to aid in the stimulation of the appropriate immune response is a critical step in the path to the development and employment of successful anti-schistosome vaccines. Here we propose a candidate vaccine based on chitosan nanoparticles associated with the antigen SmRho and coated with alginate, as an alternative strategy to induce protection against *S. mansoni* infection. This vaccination strategy offers many technical advantages, including the possibility of administration by oral route, which makes the vaccine safer than injectable vaccines and facilitates its use mainly in underdeveloped areas. Chitosan nanoparticles were prepared and characterized; the results showed that the formulation has features suitable to be delivery orally. Immunization studies suggest that the combination of chitosan nanoparticles associated to the antigen SmRho and CpG is an efficient vaccine candidate against schistosomiasis, which was able to modulate the granuloma area, that represents the major pathological response in schistosomiasis and also to induce protection against infection of *S. mansoni*.

## Introduction

Schistosomiasis remains one of the most prevalent diseases in the world and so a significant public health problem, especially in developing countries [1]. This parasitic disease affects more than 240 million people worldwide, with a further 700 million individuals living at risk of infection [2] and it causes up to 250000 deaths per year [3].

Current schistosomiasis control strategies are mainly based on chemotherapy but, despite decades of mass treatment, the number of infected people remains constant [4]. Extensive endemic areas and constant reinfection of individuals, together with poor sanitary conditions in developing countries, make drug treatment alone inefficient [5]. Many consider that the best long-term strategy to control schistosomiasis is through immunization combined with drug treatment [6]. An anti-schistosomiasis vaccine that induces even a partial reduction in worm burdens could considerably reduce pathology and limit parasite transmission [7].

The current schistosoma vaccine candidates prove not to be the most effective, so it is important to identify new antigens and to explore alternative vaccination strategies, including new adjuvants to improve vaccine efficacy [8]. In schistosomiasis, there is evidence indicating the involvement of low molecular weight proteins that bind GTP (guanosine triphosphate) during the process of maturation and deposition of eggs by the females of *S. mansoni* [9]. Over expression in female worms may be attributed to the involvement of Rho-GTPase in female reproduction processes, especially on vitelline cell maturation and/or egg laying. Immunolocalisation of *S. mansoni* Rho1 on the parenchymal cells surrounding the vitellaria adds support to this suggestion [10]. This brings an interest in understanding the role of this protein in immunological processes resulting from schistosomiasis and on the evaluation of its potential as a vaccine candidate.

Considering that schistosome infection occurs predominantly in areas of rural poverty in sub-Saharan Africa, Southeast Asia and tropical regions of the Americas [11] a candidate vaccine that could be administered by oral route could offer an economical and effective solution to mass immunization. The main advantages presented by oral vaccine delivery are the target accessibility and enhanced patient compliance owing to the non-invasive delivery method. On the other hand, for effective oral immunization, antigens and plasmids must be protected from the acidic and proteolytic environment of the gastrointestinal tract and efficiently taken up by cells of the gut associated lymphoid tissue (GALT). With this in mind, several studies have been done and showed that the association of antigens with nanoparticles increases the internalization by M cells and prevents the degradation in the gastrointestinal (GI) tract [12]. Another important aspect is that these carrier systems can act as immunostimulants or adjuvants, enhancing the immunogenicity of weak antigens [13]. Biodegradable and mucoadhesive polymeric delivery systems seem to be the most promising candidates for mucosal vaccines. Several polymers of synthetic and natural origin, such as poly(lactic-co-glycolic acid) (PLGA), chitosan, alginate, gelatin, etc., have been exploited for efficient release of mucosal vaccines and significant results have been already obtained [14].

Chitosan is the deacetylated form of chitin and has many properties suitable for vaccine delivery. It is a mucoadhesive polymer, biodegradable and biocompatible. In particular, its ability to stimulate cells from the immune system has been shown in several studies [15], [16], [17], [18]. Nevetheless, the ability of chitosan in inducing a Th1, Th2 or mixed responses is still controversial as also the type of immune response induced by different administration routes [19], [20]. Additionally, chitosan is a cationic polymer, easily form complexes or nanoparticles in aqueous medium with the possibility to adsorb proteins, antigens and DNA [21] [22] that may protect them from degradation [23]. The oral administration of antigen adsorbed nanoparticles is demanding as processes like rapid antigen desorption from the particles or the attack of the antigens by enzymes or acidic substances from the GI fluids may occur. These obstacles may be overcome by coating those antigen loading particles with an acid resistant polymer, like sodium alginate [24].

Alginate coated chitosan nanoparticles was recently described [24] and it has the particular advantage of being constructed under very mild conditions (aqueous medium and mild agitation), which is a great benefit for the encapsulation of proteins, peptides and antigens. Moreover, Borges and co-workers [25] have demonstrated that these coated nanoparticles were able to be taken up by rat Peyer's patches which is one of the essential features to internalize, deliver and target the intact antigen to specialized immune cells from the gut associated lymphoid tissue (GALT) [26].

Herein, we proposed to evaluate *in vitro* characteristics of chitosan nanoparticles associated to protein, for which the antigen Rho1-GTPase of *S. mansoni* was chosen, to be used as a candidate oral vaccine against schistosomiasis. Once *in vitro* characterization showed favorable data, its *in vivo* role was evaluated through mouse immunization. Added to that, chitosan was evaluated not only for its performance as a delivery system but also for its contribution due to its adjuvant properties.

Additionally, since a mixed Th1/Th2 response seems to be optimal for a schistosomiasis vaccine, then bacterial CpG motifs (which induce the production of IL-12 by DCs and macrophages that express the appropriate TLR95) can be used as adjuvants to boost immunity and also with the aim of inducing a TH1-like immune response that can prevent the normal Th1 to Th2 transition. With this in mind we investigated the co-administration of synthetic unmethylated oligodeoxynucleotides containing immunostimulatory CpG motifs (CpG ODN), a TLR-9 ligand, with chitosan nanoparticles.

With this in mind, on this work we will report a new strategy of vaccination against schistosomiasis based on chitosan nanoparticles associated to SmRho antigen plus the adjuvant CpG, and coated with sodium alginate.

## Materials and Methods

### Materials

Chitosan (CH) (Chimarin DA 13%, apparent viscosity 8 mPa.s) was supplied by Medicarb, Sweden. CH was purified by filtration of an acidic chitosan solution and subsequent alkali precipitation (1 M NaOH). The purified polymer was characterized by gel permeation chromatography (GPC) and Fourier Transform-Infrared Spectroscopy (FT-IR). The average weight molecular weight of the material was found to be 1.2×10^5^ (GPC in 0.5 M CH_3_COOH - 0.2 M CH_3_COONa, 25°C). The degree of acetylation determined by FT-IR according to Brugnerotto et al. [27] was found to be 16%. Endotoxin levels of the purified chitosan extracts were assessed according to Nakagawa et al. [28] using the Limulus amebocyte lysate (LAL) QCL- 1000 assay (Cambrex) and found to be lower than 0.1 EU/mL, respecting the US Department of Health and Human Services guidelines [29] for implantable devices. Imidazole modified chitosan (CHimi) was prepared as previously described [30] and it was used to prepare DNA chitosan particles, with the aim of obtaining higher rates of transfection, as reported by Moreira and co-workers [31] and also by our group [31]. Low viscosity pharmaceutical grade sodium alginate was kindly donated by ISP Technologies Inc., Surrey, UK). Class C, CpG ODN (2395) (5′-tcgtcgttttcggcgc:gcgccg-3′) was purchased from InvivoGen (San Diego, CA, USA). All the others reagents used were of analytical grade.

### Cloning and sequencing of SmRho cDNA

The SmRho cDNA sequence was amplified from an adult extract worm cDNA using specific oligonucleotides (Table 1) designed and used in a PCR reaction to amplify the complete open reading frame of SmRho (GenBank accession number AF140785). PCR was performed using Platinum Pfx enzyme (Invitrogen, Carlsbad, USA); the reaction was initiated with one cycle of 2 min at 94°C, followed by 25 cycles of 15 s at 94°C, 30 s at 56°C, and 1 min at 68°C and finalized with a step of 68°C of 3 min. PCR products were cloned by a BP recombination reaction into pDONR 221 cloning vector (Invitrogen, USA), according to manufacturer specifications. After producing the entry clone, a LR recombination reaction was performed with pDONR-rSmRho and pET-DEST42 to clone the full-length cDNA sequence of rSmRho into an expression vector (Invitrogen, USA). The resulting clone was then sequenced to confirm its identity.

**Table 1:** Oligonucleotides design to amplify the cDNA clone coding SmRho sequence.

Oligonucleotide	Sequence	Coordinates
RHO1F	5′GGGGACAAGTTTGTACAAAAAAGCAGGCTTCGAAGGAGATAGAATGGCGAGTGCGGTACG 3′	Forward
RHO1R	5′GGGGACCACTTTGTACAAGAAAGCTGGGTCAATTAAATCACACCTCCTCCTCTT 3′	Reverse

The *attB* sites, required for cloning in Gateway System, are underlined.

### Expression and purification of recombinant SmRho

To produce a recombinant (r) SmRho, the full-length DNA sequence was cloned into the expression vector pDEST42 (to produce a protein that contains a C-terminal hexahistidine tag). The resulting plasmid was transformed into *Escherichia coli* BL21 pRARE for protein expression. The recombinant protein was then purified using HiTrap Chelating HP according to the manufacturer instructions (Amersham Biosciences, Uppsala Sweden). The protein purity was assessed using sodium dodecyl sulfate polyacrylamide gel electrophoresis (SDS-PAGE).

### Evaluation of the immunogenic role of SmRho protein

After obtaining the purified protein SmRho, we wanted to confirm the ability of protein to be recognized by and to active the immune system after parasite infection. For this propose, sera of infected patient that had received drug treatment or not, compared to non-infected patient serum, were pooled and tested, by ELISA and Western Blotting. In this last assay, soluble extracts of schistosomula, eggs (SEA – soluble egg antigens) and adult worms (SWAP – soluble worm antigen preparation) were also evaluated, together with the protein SmRho, about their recognition against the sera tested.

### Preparation of coated SmRho-chitosan nanoparticles

The preparation of this delivery system contains three main steps, the manufacturing of the chitosan particles, their recombinant protein loading by adsorption and finally the coating with sodium alginate.

### Preparation of chitosan nanoparticles

Nanoparticles were prepared by mixing, while vortexing, equal volumes of a 0.1% (w/v) of chitosan in 5 mM CH_3_COONa buffer,pH 5.5 and a solution containing 0.625% (w/v) of Na_2_SO_4_, both previously heated at 55°C for 10 min. Nanoparticles were allowed to form overnight under stirring. In the following day, the suspension was centrifuged at 3200 g for 20 min at 18°C. The particles were resuspended in about 1/8 of the volume with ultrapure water (Milli-Q, Millipore).

### Loading of the particles with SmRho protein

The loading was done as described by Borges et al [24], with some adaptations. Briefly, the solution of SmRho protein (1 µg/µL) was incubated with chitosan particles under mild agitation at 18°C. The loading efficacy of the uncoated particles were calculated by an indirect way, quantifying the protein that remained in solution. After 1 h of incubation, an aliquot of the particle suspension was centrifuged at 15700 g for 30 min and the protein in supernatant was quantified by BCA-protein assay (PIERCE, Rockford, USA) using a microplate reader with a 590 nm filter (Multiskan-EX 355). The absorbance reading value was corrected subtracting the average absorbance reading obtained in the BCA-protein assay from that one of the supernatants of unloaded nanoparticles prepared exactly in the same conditions. The corrected OD value was then used to calculate the concentration using the standard curve prepared at same time from individual protein standards.

The drug loading efficiency (LE) were calculated from the following equation:
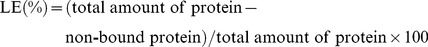
(1)


### Coating of the nanoparticles with sodium alginate

The coating was performed as described by Borges and colleagues [24]. While vortexing, equal volumes of protein-loaded particle suspension and 1% sodium alginate solution. The suspension of the particles was maintained under agitation with a magnetic stirrer for 20 min at 18°C. The suspension was then centrifuged for 30 min at 370 g and the supernatant was discarded. To chemically cross-link the alginate at the particle surface, the particles were re-suspended in 0.524 mM CaCl_2_ solution and kept under agitation for another 10 min.

The evaluation of the protein desorption during the coating procedure was performed during the incubation of the particles with sodium alginate. Aliquots of the particle suspension were collected, centrifuged at 15700 g for 30 min and the protein in the supernatant was assayed with a BCA protein assay as described previously.

### Preparation of coated SmRho-CpG-chitosan nanoparticles

SmRho+CpG ODN loaded alginate coated chitosan NPs were prepared by adding the CpG and protein at same time during the loading process and before coating the nanoparticles. The loading of CpG with chitosan particles was assessed by electrophoresis in 1% (w/v) agarose (Cambrex) gel, with 0.05 µg/mL of ethidium bromide (Q-BioGene) and also by measuring the OD of the nanoparticle supernatants at 260 nm and calculate CpG by the difference. To eliminate background interference, the supernatant of unloaded particles were treated of the same way.

### Characterization of the nanoparticles

#### Size and zeta potential determinations

To characterize the size and zeta potential, suspensions of particles loaded with SmRho protein were prepared as described above, with and without coating, and diluted in ultrapure water (Milli-Q, Millipore) to a final volume of 1 mL. The zeta potential and mean hydrodynamic size of the nanoparticles were assessed using a Zetasizer Nano ZS (Malvern, UK). The Smoluchowski model was applied for determination of the zeta potential, and cumulant analysis was used for mean particle size determination. All measurements were performed in triplicate at 25°C.

#### *In vitro* release studies

The protein release from coated particles was performed in simulated intestinal fluid (SIF) and simulated gastric fluid (SGF). The nanoparticle suspensions were added (1∶4) to individual tubes containing the release medium previously equilibrated at 37°C and placed in a shaker bath adjusted to 100 rpm. At appropriate time intervals, samples from each tube were collected and centrifuged for 15 min at 15700 g. The protein in supernatant was assayed with a BCA-protein assay. For each time evaluated, the samples were prepared in triplicate and a coated blank (without protein) nanoparticles suspension was submitted to the same conditions and was used as a blank for correction on the BCA-protein assay. In addition, non-bound protein in the alginate coated particle suspension was determined by BCA-protein assay in order to calculate the initial quantity of protein encapsulated in the beginning of the *in vitro* release study.

### Immunization studies

#### Mice and parasites

Six-week-old female C57BL6 mice were purchased from the Federal University of Minas Gerais (UFMG) animal facility. Animals had free access to food and water, with 12 h light/dark cycle.

*S. mansoni* cercariae were maintained routinely on Biomphalaria *glabrata* snails at the Center of Research Rene Rachou-Fiocuz (CPqRR). For infections, cercariae were prepared by exposing infected snails to light for 1 h to induce shedding. Cercariae numbers and viability were determined using a light microscope prior to infection.

#### Treatment groups

Six groups (n = 5) were submitted to different treatments, as is described in Table 2. In order to evaluate the adjuvant effect of chitosan, nanoparticles without protein were prepared (Group II). This property was also evaluated in the group VI which the antigen was administered, by intramuscular route, adsorbed in chitosan nanoparticles and coated with alginate, but without adding any other adjuvant commonly used. Coated SmRho-chitosan nanoparticles were also prepared with adding the adjuvant CpG in order to verify if it could potentiate the induced immune response and these nanoparticles were administered in groups V.

**Table 2:** Treatment groups and the formulation administered to each one.

Administration route:	Oral	Oral	Oral	Oral	Oral	Intramuscular
**Groups (n = 5)**	I	II	III	IV	V	VI
**PBS**	X		X			
**Chitosan NPs** *		X				
**SmRho (solution)**			50 µg			
**SmRho loaded alginate coated chitosan NPs**				50 µg		50 µg
**SmRho-CpGODN loaded alginate coated chitosan NPs**					50 µg+10 µg	

* NPs: Nanoparticles.

### Immunization schedule and collection of samples

The different formulations were administered orally with a gavage-feeding needle (groups I–V) and intramuscularly with an injection in the tibialis anterior muscle (group VI). The primary immunization was followed by two immunizations with an interval of two weeks. Seven days after the last immunization mice were challenged and then, after 50 days mice were sacrificed.

Blood samples were collected from tail veins from mice of each experimental group at two-week intervals and the sera were prepared by centrifugation and stored at −20°C until further analysis. Serum was collected from immunized and control mice to measure kinetics of SmRho specific antibodies.

### Measurement of specific anti-SmRho antibodies

A measurement of specific anti-SmRho antibodies was performed using an indirect enzyme-linked immunoabsorbent assay (ELISA) as described elsewhere [32]. Briefly, maxisorp 96-well microtiter plates (Nunc, Roskilde, Denmark) were coated with 10 µg/mL of SmRho in carbonate-bicarbonate buffer, pH 9.6, for 16 h at 4°C, followed by washes and then blocked for 1 h at 37°C with 200 µl/well of PBS-casein (phosphate buffer saline, pH 7.2 with 1.6% of casein). Next, 100 µl of serum sample from individual mice diluted 1∶100 in PBS-casein was added to each well and was incubated for 1 h at 37°C. Plate-bound antibodies were detected by peroxidase-conjugated anti-mouse IgG (SIGMA), IgG1 and IgG2a (Southern Biotechnology Associates, Inc., Birmingham, AL, USA) diluted 1∶5000 in PBS with 0.25% casein. The plates were revealed by the addition of 100 µl of detection solution (R&D systems, Minneapolis, USA) containing tetramethylbenzidine (Thermo Scientific Pierce) and H_2_O_2_ in each well; after 20 min reactions were stopped with the addition of 50 µl of 5% (v/v) of sulfuric acid per well. The absorbance was read at 450 nm in an ELISA plate reader (ELX 800 BIO-TEK Instruments Inc.).

### Challenge infection and worm burden assessment

Seven days after the last immunization, mice were challenged through percutaneous exposure of abdominal skin to water containing 25 cercariae (LE strain) for one hour. 50 days after challenge, mice were sacrificed and adult worms were perfused from their portal veins [33]. Protection was calculated by comparing the number of worms recovered from each vaccinated group compared with control group using the following equation:
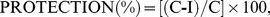
(2)where C represents the worms recovered from the saline control group and I represents the worms recovered from the experimental group.

### Cytokine analysis

#### Antigen preparation

Antigenic preparations were obtained from schistosome eggs and adult worms, prepared as soluble supernatant fluids from buffered saline homogenates of the respective life-cycle stages [34]. Cytokine production was evaluated when splenocytes were stimulated with rSmRho, SWAP or SEA.

#### Cytokine experiment

Cytokine experiments were performed using splenocyte cultures from individual mice of control and experimental groups. Splenocytes were isolated from macerated spleens of individual mice at day 50 post-infection. Cells were washed twice with sterile PBS and were plated at a concentration of 1×10^6^ cells per well in RPMI 1640 medium (Gibco, Carlsbad, CA, USA) supplemented with 10% FBS, 100 U/mL of penicillin G sodium, 100 µg/mL of streptomycin sulfate, and 250 ng/mL of amphotericin B. Splenocytes were maintained in culture with medium alone or stimulated with SmRho (25 µg/mL), SWAP (25 µg/mL) or SEA (25 µg/mL). The 96-well plates (Nunc, Denmark) were maintained in an incubator at 37°C with 5% CO_2_. Culture supernatants were collected after 72 h for IL-10, TGF-β and IFN-γ analysis. IL-10, IFN-γ and TGF-β concentrations were measured using Duoset ELISA kit (R&D Systems, USA) according to the manufacturer instructions.

### Hepatic granuloma analysis

Liver fragments from mice (5 mice per group) of control and experimental groups immunized and infected, were collected 50 days post-infection in order to evaluate the effect of immunization on granuloma formation. Livers fragments were fixed in 10% paraformaldehyde. Fragments processed for paraffin embedding and histopathological sections were cut using a microtome at 5 µm. Sections were stained on a slide with hematoxilin-eosin (HE). The areas of individual granulomas were obtained through the MacBiophotonics ImageJ software analyzer. Fifteen granulomas from each mouse with a single well-defined egg were randomly chosen using a microscope with the 4× objective lens; granulomas were then scanned using JVC TK-1270/RGB microcamera. Using a digital pad, the total area of each granuloma measured, and the results were expressed in square micrometers.

### Statistical analysis

Statistical analysis was performed using the ANOVA test using the Graph Pad Prism 5 software package. The Bonferroni test was used to compare subgroups with the level of significance set at p<0.05.

### Ethics statements

The studies regarding sera samples taken from patients were approved by Research Ethics Committee (COEP) of UFMG, the protocol number 523/07, and a written informed consent was obtained from each patient before blood collection. Experiments with animals were performed in compliance with the guidelines of the Institutional Animal Care and Committee on Ethics of Animal Experimentation (“Comitê de Ética em Experimentação Animal” – CETEA, national guidelines, Law number 11.794, 8/10/2008) from Universidade Federal de Minas Gerais (UFMG); protocol number 204/2009 was approved on 24/03/2010.

## Results

### Cloning, expression and purification of recombinant SmRho

The full-length sequence of the *S. mansoni* cDNA encoding SmRho was obtained from an adult worm cDNA library using PCR with specific oligonucleotides. The resulting full-length cDNA displayed an ORF of 579 bp, encoding a protein of 193 amino acids with a predicted molecular mass of approximately 21.8 kDa and an isoelectric point of 5.70. BlastP comparisons of the deduced protein sequence in GenBank exhibit a complete identity and similarity to *S. mansoni* Rho-GTPase protein (EMBL-Bank CDS: AAD31508.1)(data not shown).

**Figure 1:**
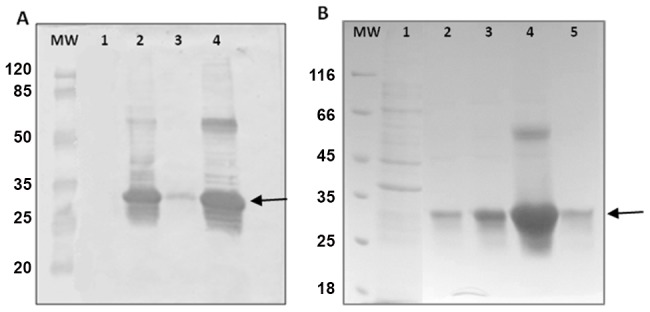
Expression and purification of rSmRho. (A) Western Blotting profile of the *E. coli* (BL21 pRARE) transformed with the pDEST42-SmRho. Lane: MW – Prestained protein molecular weight marker (Fermentas). Lanes 1 and 2 represent a clone before and after induction with 0.5 mM IPTG, respectively. Lanes 3 and 4: soluble and insoluble fraction after *E. coli* (BL21 pRARE) lysis, respectively. (B) SDS-PAGE 12% profile of Ni^2+^ chromatography. Lanes: MW – Unstained protein molecular weight marker (Fermentas); 1 - flow through; 2–5 fractions of rSmRho–6×HIS-tag fusion protein eluted after Ni2+ chromatography. Positions of molecular mass standards (kDa) are indicated. Arrows indicate the purified rSmRho.

The nucleotide sequence of this protein was cloned into a pET-DEST42 expression vector and the protein was expressed in *E. coli* BL21 pRARE strain. Protein extract of transformed bacteria showed a band at ∼26 kDa when induced with IPTG, since the plasmid add a C-terminal hexahistidine tag in the protein expressed. The bacteria were then lysed and the lysate separated into soluble and insoluble fractions. The inclusion bodies were shown to contain the majority of the recombinant protein (Figure 1A), which was mostly solubilized by extraction with 0.9% (w/v) N-Laurylsarcosine. The protein was bound to a nickel-charged column under denaturing conditions, and purified by affinity chromatography through an imidazole linear gradient. Eluted fractions containing rSmRho were pooled, and protein yield after purification was estimated to be around 3 mg/L (Figure 1B). After buffer exchange the protein was used in further experiments.

### Recognition of SmRho by sera of patients infected with *S. mansoni*

**Figure 2:**
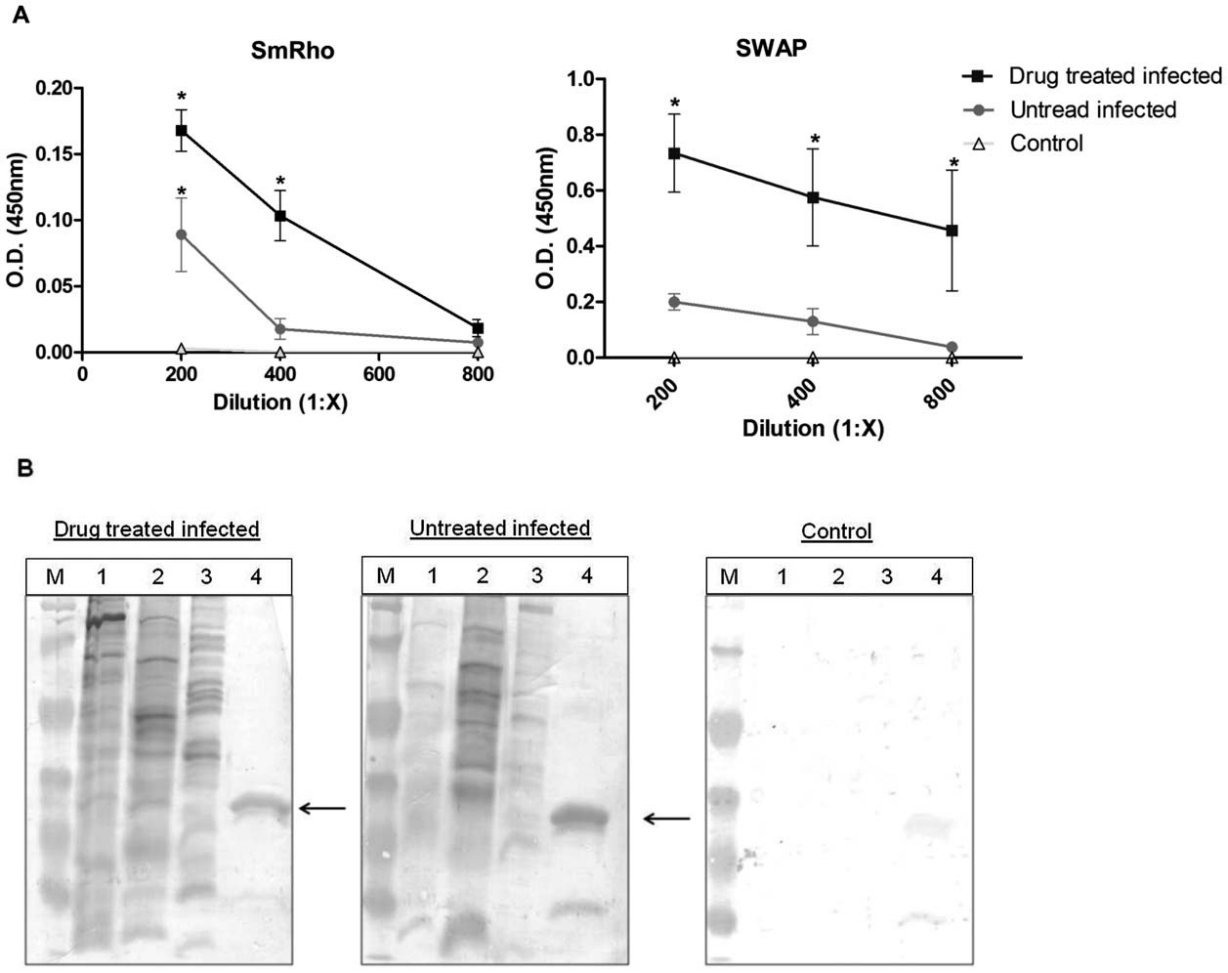
Reactivity of sera from control and *S. mansoni* infected patient, drug treated or untreated, against SmRho and SWAP observed by ELISA^#^ (A) and against SmRho, SWAP, SEA and schistosomula extract by Western Blotting (B). M: Standard molecular weight Page Ruler Unstained Protein Ladder (Fermentas), 1: SWAP; 2: SEA, 3: schistosomula antigens; 4: recombinant protein Rho1-GTPase de *S. mansoni*. ^#^The results are presented as the mean absorbance measured at 450 nm. Statistically significant differences of sera from infected patient with the control group, non infected patient, in each sera dilution evaluated, are indicated by (*) for p<0.05. The molecular weight markers, from top to bottom, are: 116, 66, 45, 35, 25, and 18 kDa.

To confirm the immunogenicity of SmRho in *S. mansoni* infection, the recognition of the protein by sera of infected patients, who had received drug treatment or not, was evaluated. The results obtained in the ELISA test showed that SmRho was not recognized by sera of control patients, i.e. not infected with *S. mansoni*. On the contrary, sera of infected patients, in particular of drug treated ones, showed a specific reaction against SmRho (Figure 2A). Additionally, the immunogenicity of SmRho was evaluated by Western blot assay corroborating the above-mentioned result (Figure 2A). It was observed again the reactivity of the protein SmRho with sera of infected patients, drug treated and untreated, and no reaction in the control group. In the membrane incubated with sera of treated infected patient a protein band with a molecular weight close to SmRho band in the soluble extract of eggs (SEA) and adult worms (SWAP) was observed as well. This higher reactivity in treated group is probably due to a greater exposure of the protein after drug treatment which increases the recognition by immune system cells. Sera types incubated with each membrane are represented in Figure 2B.

### Characterization of nanoparticles

The preparation of the delivery system was performed as described by Borges and colleagues [24] and it contains three main steps, the manufacturing of the chitosan particles, their protein loading by adsorption and finally the coating with sodium alginate. The efficiency of this process was assessed by the quantification of non-bound protein that remained in the supernatant after the loading and coating steps. In Table 3 one can observe that the encapsulation efficiency of the protein in the chitosan nanoparticles before the coating with alginate was around 95%. After the coating around 76% of the protein remained bound to the particles.

**Table 3:** SmRho loading efficiency on chitosan particles before and after coating with alginate.

	Loading Efficiency (mean ± SD)
Before coating with alginate	95.5%±0.9
Alginate coated chitosan-SmRho particles	76.6%±3.1

The association of CpG to alginate coated chitosan-SmRho nanoparticles was evaluated by two techniques using the supernatants of the particles. Both techniques indicated a CpGODN loading efficiency of about 100% since no DNA mobility in electrophoresis was verified as well as no absorbance at 260 nm was detected (data not shown).

The size of chitosan-SmRho particles, before and after coating, was determined by light scattering technique. One of the major currently described drawbacks of this methodology of particle preparation is the high polydispersity of the obtained nanoparticles [35] that results from particle aggregation during its formation. The average size presented by particles was approximately 750 nm scale before coating and after that a reduction of particles size was observed (Table 4). The unpredictable result of coated nanoparticles had a smaller size than those uncoated was most probably related with the aggregation phenomena of the particles. Therefore, average size values had the contribution of aggregates size, presented on particles suspension.

**Table 4:** Size and zeta potential of chitosan particles before and after coating.

	Size (nm) mean ± SD	Polydispersity (PdI)	Zeta potential (mV) mean ± SD
Before coating with alginate	743.3±107.6	0.550±0.116	33.5±2.7
Alginate coated chitosan-SmRho particles	435.0±119.4	0.475±0.096	−38.6±3.8

These particles were also characterized in terms of zeta potential (Table 4). In the first step, zeta potential determinations have shown that an excess of polymer allowed the assembly of particles with a positive global net charge. During the coating procedure an inversion of the surface charge of the particles to negative values was observed due to the negative charge of sodium alginate. This zeta potential inversion is a strong indication of the presence of an alginate coating on the surface of the particles.

### *In vitro* release studies

**Figure 3:**
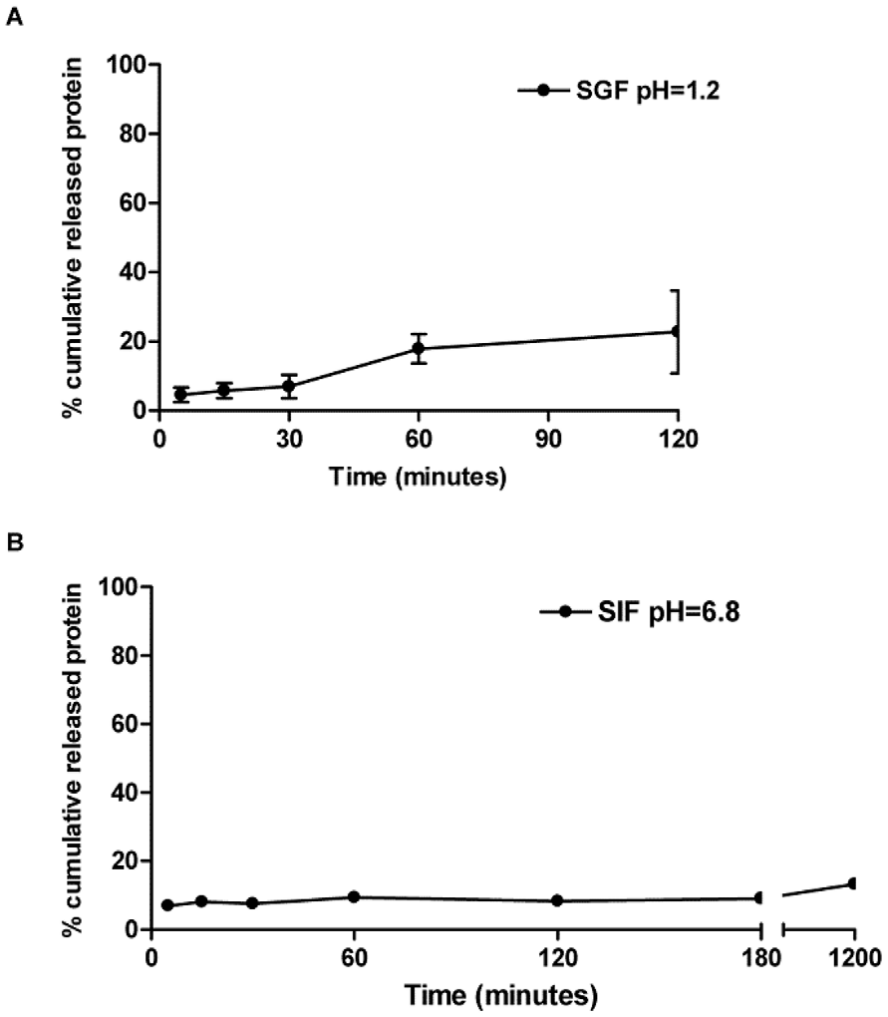
Release profile of protein SmRho from alginate-coated chitosan nanoparticles in SGF (A) and SIF (B) at 37°C (mean ± SD, n = 3).

Release studies were performed to evaluate the stability of the coated nanoparticles and the profiles of SmRho protein desorption from these nanoparticles in simulated gastric fluid (SGF) and simulated intestinal fluid (SIF), at 37°C. It can be observed in Figure 3 that coated nanoparticles presented a great stability in SGF, with less than 40% of the protein released after 2 hours and it was even better in SIF, where less than 15% of the protein was released after 20 hours of assay.

### Antibody profile following mice immunization

**Figure 4:**
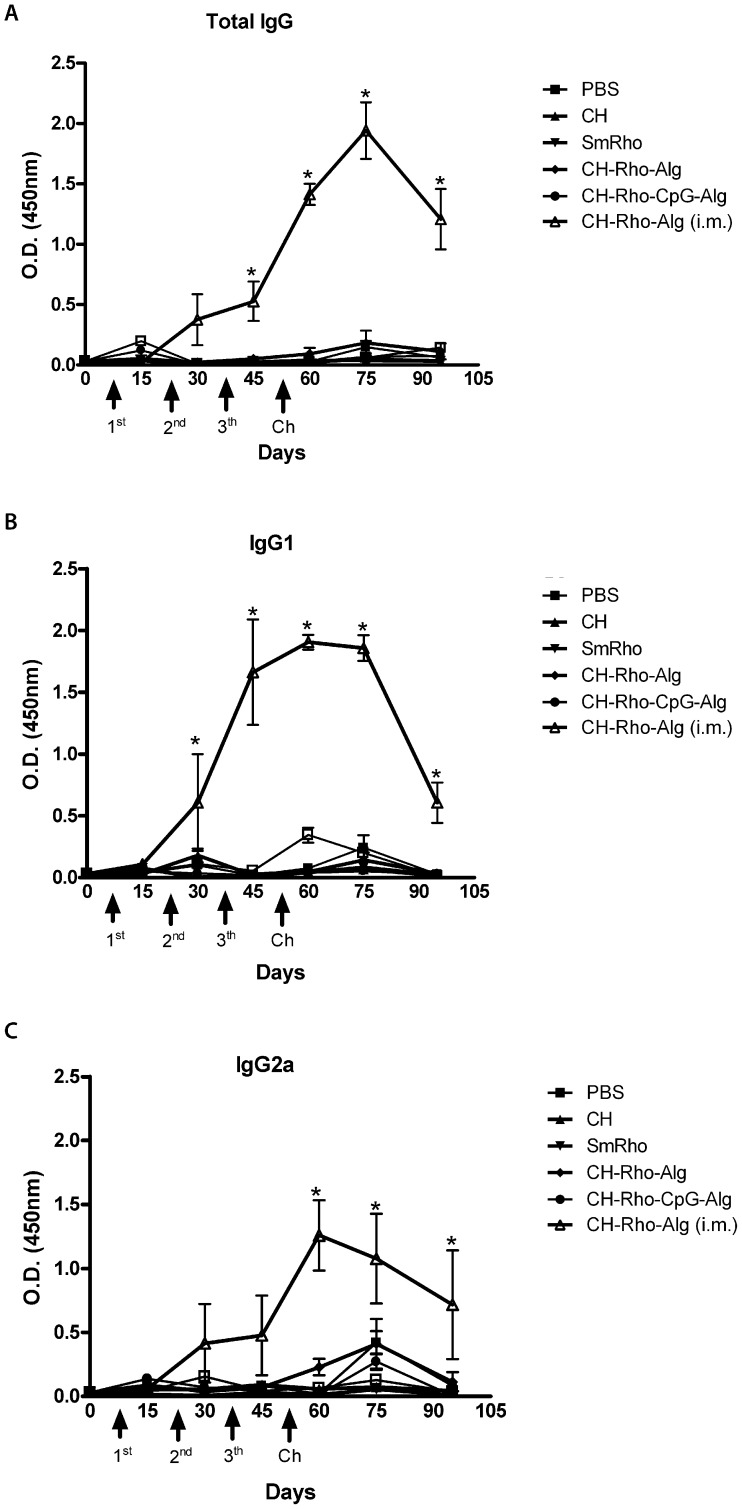
Serum anti-SmRho antibodies collected from mice of all treatment groups was measure for total IgG (A), IgG1 (B) and IgG2a (C) levels. Sera of immunized mice were collected at days 15 (one week after the first immunization), 30, 45, 60, 75 and 95 and assayed by ELISA. The results are presented as the mean absorbance measured at 450 nm for each group. The arrows indicate the time of prime-boost immunization and the challenge infection (Ch). Statistically significant differences of vaccinated mice compared with the control group, PBS, in each time evaluated, are indicated by (*) for p<0.05.

Further *in vivo* studies were performed to investigate the potential utility of rSmRho loaded chitosan-based nanoparticles in eliciting the production of antibodies or modulating the immune response following intramuscular or oral administration of the suspension of the particles. To evaluate the levels of SmRho-specific IgG antibodies serum samples from vaccinated animals from each group were tested by ELISA. The measures of IgG antibodies showed that nanoparticles were able to induce the production of specific SmRho antibodies mainly in the experimental group vaccinated with coated SmRho-chitosan nanoparticles by intramuscular route, which showed high levels of IgG that appeared at the day 45 (5 weeks after the first immunization) and presented the highest level at the day 75, compared to the control group (Figure 4). To determine the type of immune response induced after vaccination, the subclasses IgG1 and IgG2a were also analyzed. For this same experimental group, the levels of specific anti-SmRho IgG1 antibodies were increased since day 30 until day 90, when the animals were sacrificed, and a peak of IgG1 antibodies was observed at the day 60, after performed the three rounds of immunization. The levels of IgG2a were also increased after the three rounds of immunization, with a peak at the day 60, which remained elevated until the day 90. The high levels of IgG1 and IgG2a showed a mixed Th1/Th2 profile. In relation to the remaining groups, there was no significant production of specific SmRho antibodies during the period evaluated.

### Protective effect of vaccination

To investigate the protective activity induced by vaccination with different formulations of chitosan nanoparticles in murine model of *S. mansoni* infection, immunized mice were challenged with 25 cercariae. The difference in the number of adult worms recovered in the experimental groups compared to control group was calculated 50 days post-challenge (Table 5). Groups immunized with coated SmRho nanoparticles, associated or not to CpG also presented the highest level of protection protection of 48% and 55%, respectively. Interesting, the group of animals immunized with CH nanoparticles without SmRho protein and challenged with cercariae showed a significant reduction of 47% in adult worm burden. This result suggests that chitosan has an important role in inducing nonspecific immunity against *S. mansoni* infection, and that the adjuvant CpG did not have a considerable contribution in reducing worm burden in this experiment.

**Table 5:** Protective effect and liver granuloma size induced by C57BL/6 mice vaccination and challenged with 25 *S. mansoni* cercariae.

Groups	Total worms Mean ± SD	Percentage reduction of worm burden	Hepatic granuloma area (µm^2^) Mean ± SD	Percentage reduction of granuloma area
PBS (group I)	19.0±2.4	-	112501±49914	-
Chitosan NPs (group II)	10.1±0.81*	47*	83269±50226	26.0
SmRho (group III)	14.7±3.1	22.8	85076±60937	24.3
CH-SmRho-Alginate (group IV)	8.5±1.3*	55*	80473±40649	28.5
CH-SmRho-CpG-Alginate (group V)	10.0±4.2*	48*	69282±57918	38.4*
CH-SmRho-Alginate (i.m.) (group VI)	13.0±2.0	31	86715±67237	22.9

* **Statistically significant compared with the control group (p<0.05).**

### Coated SmRho-CpGODN chitosan nanoparticles oral vaccination induced granuloma down-modulation

To evaluate the effect of the proposed vaccine on reducing granuloma reactions, histological analysis was performed by digital morphometry. Seven days after the third immunization, mice were challenged with 25 cercariae. After 50 days of challenge infection, mice were sacrificed and liver samples were taken for histological analysis. Hematoxilin and eosin stained liver sections were then used to measure the size of individual granulomas. Vaccination with coated SmRho-CpG-chitosan nanoparticles by oral route reduced liver granuloma area by 38.4% (Table 5), compared with mice that were immunized with PBS. The remaining groups also presented a considerably granuloma area reduction, however, these granulomas were not as small as those observed in groups above mentioned. These findings suggest that the antigen SmRho associated with CpG in nanoparticles was important to induce this anti-pathological effect.

### Cytokine profiles induced by SmRho immunization

**Figure 5:**
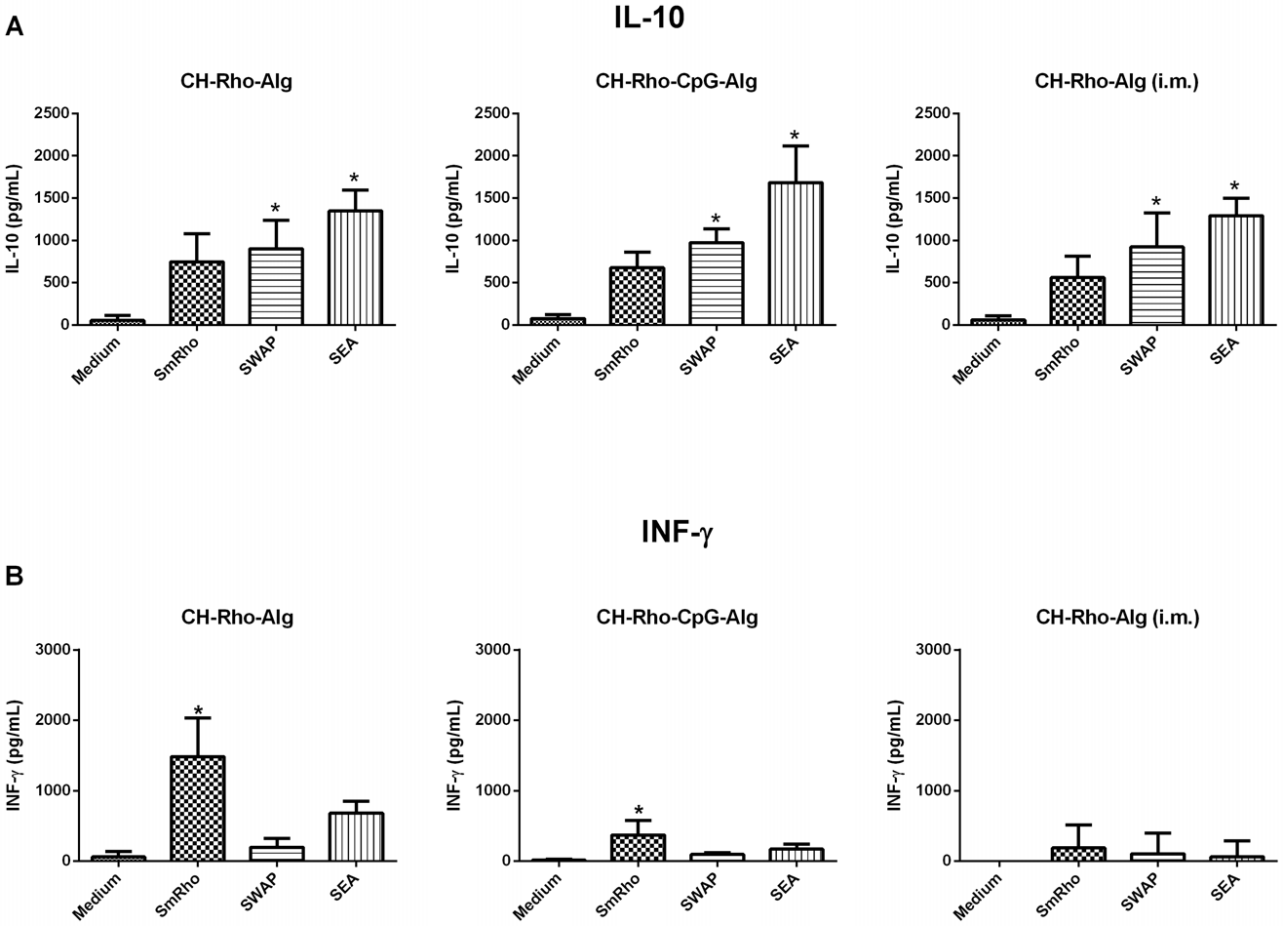
Cytokine profiles of mice immunized with coated SmRho-chitosan nanoparticles. Splenocytes isolated from mice immunized with CH-Rho-Alg, CH-Rho-Cpg-Alg, and CH-Rho-Alg (i.m.) were assayed for IL-10 (A) and INF-γ (B) production in response to *in vitro* stimulation with SWAP (25 µg/ml), rSmRho (25 µg/ml), SEA (25 µg/ml) or medium alone as control. Results represent the mean ± SD of each group. *Statistically significant differences between cytokines produced after SWAP, rSmRho or SEA stimulation compared with unstimulated splenocytes (control) (p<0.05).

Cytokine profile evaluation was performed using splenocyte cultures from individual mice immunized with chitosan-based nanoparticles. The production of IFN-γ, IL-10 and TGF-β was measured in the supernatants of spleen cells cultured only with complete RPMI medium or in the presence of SmRho, SEA (soluble egg antigens) or SWAP (soluble worm antigen preparation). The highest levels of the immunomodulatory cytokine IL-10 were produced by SEA-stimulated splenocytes from mice immunized with coated SmRho-CpG-chitosan nanoparticles, compared with the control-stimulated splenocytes. In the others experimental groups significant levels of IL-10 were also observed, although their levels were not so high compared with those above described (Figure 5A). Significant levels of IFN-γ, a cytokine typical of Th1-type immune response, were produced by SmRho-stimulated splenocytes from groups immunized with coated SmRho-chitosan and coated SmRho-CpG-chitosan (Figure 5B). Taken together, these results show that groups which presented the highest levels of the immunomodulatory cytokine IL-10, also were able to achieve a significant protective response and a reduced liver pathology, which is probably related to the prevention of an excessive Th1 and/or Th2 response by IL-10.

## Discussion

Schistosomiasis is one of the most important neglected tropical diseases and an effective control is unlikely in the absence of improved sanitation and vaccine. The antigens tested, so far as vaccine candidates, prove not to be so effective as desirable, consequently, it is important to continue identifying new target antigens [36].

The selection of a suitable delivery system and an adjuvant to aid in the stimulation of the appropriate immune response is a critical step in the path to the development and employment of successful anti-schistosome vaccines, and a number of approaches are being tested, with some success [8].

Here we proposed a candidate vaccine formulation based on SmRho antigen loaded chitosan nanoparticles, coated with alginate, as an alternative strategy to induce protection against *S. mansoni* infection. This vaccination strategy offers many technical advantages, including the possibility of administration by oral route, which makes the vaccine safer than injectable vaccines and facilitates its use mainly in underdeveloped areas [37].

The recombinant expression of SmRho was optimized using an *E. coli* pRARE lineage, which co-expresses rare tRNAs required for the synthesis of some eukaryotic proteins. The expression and purification of the protein of interest was obtained with high yield and showed a protein with approximately 26 kDa of molecular mass. The immunogenicity of SmRho protein was confirmed through the reaction with sera of patient infected with *S. mansoni*. Besides that, the presence of SmRho in soluble egg and adult worms extracts was verified, which is supported by Vermeire and co-workers reports [10].

The methodology for the preparation of coated chitosan nanoparticles was successfully adapted from Borges and co-workers [24] as demonstrated by particle characterization results. The protein loading of the nanoparticles was done by adsorption process based on electrostatic interaction [38] and this process was favored considering that SmRho has an isoeletric point of 6.5. Consequently, in a physiological solution that has a pH of 7.4, SmRho is negatively charged and can easily interact with the positively charged chitosan nanoparticles. In the present work, SmRho loading efficiency of uncoated particles was 95%, which are better than those results found in literature [39] [24]. After coating, the loading efficiency showed a significant decrease to 76%. Nevertheless, the result obtained here was still better than those found by Borges and co-workers [24] and Li and co-workers [39], which was 60% and 66%, respectively, for ovalbumin. A disturb of the protein adsorption equilibrium can occur and a new equilibrium can be established having alginate as a direct competitor for positive charges on chitosan surface which explain our results.

*In vitro* release studies were performed in SGF and SIF medium at 37°C in order to evaluate the release profiles of protein SmRho and also to assess the stability of the designed delivery system when submitted to medium with different pH, ionic strength at physiological temperature. In SGF, there was a gradual release of the protein, and after 2 h, about 30% of the protein had been released. After this period, it is believed that the particles have passed through the stomach therefore, it can be deducted that nanoparticles are quite resistant to the influence of acid environment. Despite using a similar system, Borges et co-workers (2006) [25] obtained a protein release rate of over than 90% in SGF medium after the same period of the release test. The protein release profile in SIF had an even better result, since after 20 h of assay, only about 15% of the protein had been released. This result was also better than that obtained by Borges and colleagues [25] and Li and colleagues [39]. Again, the system under study remained stable in SIF medium conditions, what is desirable for an efficient antigen delivery. After characterization and based on the results which showed that the nanoparticles have suitable features to be delivery orally, the immunization was realized to investigate the effect of coated chitosan nanoparticles loaded with antigen on mice immune system and their potential to prevent infection of *S. mansoni*. Our results demonstrated that an anti-pathological or a protective response induced against infection with cercarie are not necessary correlated with high levels of specific antibodies. This can be presumed because only the group intramuscularly immunized with coated chitosan nanoparticles presented high levels of SmRho specific IgG1 and IgG2a antibodies, and it did not show any significant reduction in granuloma area or in worm burden. On the other hand, SmRho-CpG-chitosan nanoparticles administered by oral route reduced liver granuloma area by 38.4% and were not able to induce systemic specific antibodies. This result confirm our previous work, recently published [31] where the immunization with chitosan-DNA nanoparticles did not induce antibodies, however was able to reduced liver pathology. Nevertheless, this group (CH-Rho-Alg i.m.) showed an important role of alginate coated chitosan nanoparticles as adjuvant, considering that any other adjuvant or immunopotentiator was used and high levels of antibodies were produced.

It is a consensus that the development of new and safe adjuvants is necessary not only for parenteral vaccination but also for the more challenging mucosal routes of administration in order to maximize the effectiveness of new antigens as well as those already available [40]. Within this perspective, and due to their unique and interesting properties recently reviewed in several scientific journals [41], [42], [43], chitosan-based nanoparticles have been used associated with various antigens as carrier system for vaccination and administered by different mucosal routes [12], [44], [45]. However, its potential as an adjuvant to parenteral vaccination has been less studied. In a previous study [16] a solution of chitosan was explored as an adjuvant for mice subcutaneous immunization with a model antigen. It was shown that chitosan was able to increase more than five times antibody titers and the proliferation of specific CD4+T cells more than six times.

With respect to sodium alginate, some studies show that alginate microparticles are internalized by M cells of the mucosa [46] and were able to transport antigens to mucosa associated lymphoid tissues, inducing systemic and mucosal immune response in a variety of animal species following oral administration [47]. Moreover, alginate microparticles can be used not only as a carrier system, but also as an adjuvant, as it was shown to induce the production of cytokines such as TNF-α, IL-6 and IL-1 [48] and it increases the antibodies production similarly to other adjuvants, such as incomplete Freund's adjuvant and aluminum hydroxide [47]. Mata and colleagues also showed that immunization studies in Balb/c mice by intradermal route using alginate as adjuvant elicited higher humoral and cellular immune responses leading to more balanced Th1/Th2 profile [49].

Pathology resulting from granuloma formation around the eggs in murine schistosomiasis is characterized by Th2-type of immune response and the granuloma size can be reduced by neutralization of IL-4 [50]. Thus, morbidity and mortality in murine schistosomiasis were hypothesized to be developed as a direct consequence of the egg-induced Th2 type of immune response. Herein, we suggest that the addition of CpG to nanoparticles formulation was important to induce, even in a small proportion, a shift from Th2 to Th1 immune response and also due a regulatory role for IL-10 on CpG–ODN-induced Th1 immune response, as recently reported by Jarnicki et al [51]. They found that TLR ligands, including CpG–ODNs promoted IL-12 and IL-10 production from dendritic cells. This resulted, with both components, Th1 immune response and induction of the IL-10-secreting T regulatory cells (Treg) could help to prevent an exacerbated granuomatous reaction. The role of CpG as an immune modulator was also described by Slütter and Jiskoot [52] and the mechanism by which CpG induces a reduced granuloma formation is probably the same as observed when CpG is used in therapy for asthma/allergy as has been widely reported [53]. In the present study the group immunized with formulations CH-Rho-CpG-Alg, which showed a reduced granuloma area of 38%, also showed higher levels of the immunomodulatory cytokine IL-10 when splenocytes were stimulated with SEA. This probably contributed to reduce inflammation and liver pathology observed in this group. It has been reported that IL-10 plays a key regulatory role in preventing the development of severe pathology due to excessive Th1 and/or Th2 responses [54]. Additionally to these balance of cytokines produced in groups immunized with coated chitosan nanoparticles containing CpG, the presence of the antigen SmRho is also supposed to have an important role in the modulation of immunopathological responses of *S. mansoni* infection. This is likely due to the induction of INF- γ production that can prevent the normal Th1 to Th2 transition that occurs in infected hosts after the onset of egg production by the parasites, and therefore acts preventing the development of severe chronic morbidity [55]. These observations revealed an anti-pathological role of SmRho but more studies are required to obtain a better understanding of the involvement of this protein in the process of infection of *S. mansoni*.

To determine whether coated chitosan nanoparticles conferred protection against *S. mansoni* infection, immunized mice were challenged with cercariae and worm burdens were assessed. All groups immunized with coated chitosan nanoparticles by oral route showed a significant reduction in worm burden and even the group immunized with chitosan nanoparticles without protein showed a 47% of protection. It suggests that chitosan has an important role in inducing a protective immune response against schistosomiasis that is likely due to its immunostimmulatory properties. Nevertheless, the mechanism behind the ability of chitosan in inducing protection is still unknown. Its role is more likely related to trigger an innate immune response, since chitin, which is a progenitor to chitosan, has been demonstrated to function as a PAMP and it is linked with the accumulation of innate cells including alternatively activated macrophages, eosinophils and basophils [56] [57]. At sites of infection with chitin-containing agents anti-infectious immune responses and local chitinases are believed to induce chitin fragmentation. The ability of chitin to induce an acute inflammatory response is already described, but it seems to act by different pathways depending of the size of the fragment and the time point of assessment. Da Silva and co-workers demonstrated that chitin fragments induced a macrophage- and neutrophil-rich inflammatory response with only a modest degree of eosinophil infiltration, while Reege et al. showed mainly eosinophil-based response [17], [58]. When viewed in combination, these studies suggest that chitin induces an inflammatory response that is initially neutrophilic and becomes eosinophilic over time. In the early times, macrophages were shown to be stimulated by chitin particles *in vitro* and *in vivo*, by a TLR-2 dependent mechanism that utilizes a MyD88-dependent pathway to induce IL-17 elaboration and enhance the expression of the IL-17AR. These studies also demonstrated that this novel innate immune pathway plays an essential role in the regulation of macrophage cytokine production and the induction of acute inflammation [58]. Additionally, it is well described that IL-4/IL-13-activated alternative macrophages are essential for surviving acute schistosomiasis [59]. These facts cooperate with the convincing evidence that immune elimination of challenge parasites occurs in the lungs and macrophages is expected to mediate the protective response [60]. At later times Yasuda et al., suggested that the effects of IL-33 for the expansion and the activation of eosinophils might aid to expel infected worms from the lungs [18]. These reports support an important theory by which chitosan induce a protective immune response against *S. mansoni* infection, however it needs to be further investigated.

Mice immunized with coated chitosan nanoparticles associated or not with CpG also showed high percentage of protection 48% and 55%, respectively. The antigen and CpG seems not to have a high contribution in conferring protection against worm infection, nevertheless, they demonstrated important roles in granuloma down-modulation, as discussed before.

As a final conclusion of this work, we believe that the combination of chitosan nanoparticles associated to the antigen SmRho plus CpG is an efficient vaccine formulation candidate against schistosomiasis in light of the data obtained from murine studies, which was able to modulate the granuloma area, that represents the major pathological response in schistosomiasis and also to induce protection against infection of *S. mansoni*. It is important to highlight that these results were obtained with oral administration of the formulation and can be compared to those obtained from conventional routes of administration. Comparing with our previous work, in which DNA-chitosan nanoparticles were explored, this system achieved better results to be used as a vaccine because induced both protection as well as reduced the granulomatous reaction, while the first presented just an anti-pathological effect with granuloma modulation. Chitosan-based nanoparticles were also found to play an important role as adjuvant and this characteristic should be more explored with other antigens. Furthermore, the role of chitosan in inducing a protective immune response against schistosomiasis deserves special attention and requires more studies to confirm and understand this feature. Taken together, these results support this new strategy to find a safe and efficacious vaccine against schistosomiasis.

## References

[1] King CH (2009) Toward the elimination of schistosomiasis. New England Journal of Medicine 360: 106–109. 19129524 10.1056/NEJMp0808041

[2] WHO WHO (2010) http://www.who.int/mediacentre/factsheets/fs115/en/index.html. In: N°115 Fs, editor.

[3] van der Werf MJ, de Vlas SJ, Brooker S, Looman CWN, Nagelkerke NJD, et al (2003) Quantification of clinical morbidity associated with schistosome infection in sub-Saharan Africa. Acta Trop 86: 125–139. 12745133 10.1016/s0001-706x(03)00029-9

[4] Harder A (2002) Chemotherapeutic approaches to schistosomes: current knowledge and outlook. Parasitology Research 88: 395–397. 12049454 10.1007/s00436-001-0588-x

[5] Bergquist NR, Colley DG (1998) Schistosomiasis vaccine: research to development. Parasitology Today 14: 99–104. 17040715 10.1016/s0169-4758(97)01207-6

[6] Bergquist N (2002) Schistosomiasis: from risk assessment to control. Trends in Parasitology 18: 309–314. 12379951 10.1016/s1471-4922(02)02301-2

[7] Chitsulo L, Loverde P, Engels D (2004) Schistosomiasis. Nature Review Microbiology 2: 12–13. 10.1038/nrmicro801 15035004

[8] McManus DP, Loukas A (2008) Current status of vaccines for schistosomiasis. Clin Microbiol Rev 21: 225–242. 18202444 10.1128/CMR.00046-07 PMC2223839

[9] Schüssler P, Grevelding C, Kunz W (1997) Identification of Ras, MAP kinases, and a GAP protein in *Schistosoma mansoni* by immunoblotting and their putative involvement in male-female interaction. Parasitology 115: 629–634. 9488874 10.1017/s003118209700173x

[10] Vermeire JJ, Osman A, LoVerde PT, Williams DL (2003) Characterisation of a Rho homologue of *Schistosoma mansoni* . Int J Parasitol 33: 721–731. 12814652 10.1016/s0020-7519(03)00046-8

[11] Gryseels B, Polman K, Clerinx J, Kestens L (2006) Human schistosomiasis. The Lancet 368: 1106–1118. 10.1016/S0140-6736(06)69440-3 16997665

[12] Mishra N, Goyal AK, Tiwari S, Paliwal R, Paliwal SR, et al (2010) Recent advances in mucosal delivery of vaccines: role of mucoadhesive/biodegradable polymeric carriers. Expert Opin Ther Pat 20: 661–679. 20345332 10.1517/13543771003730425

[13] Jabbal-Gill I, Lin W, Jenkins P, Watts P, Jimenez M, et al (1999) Potential of polymeric lamellar substrate particles (PLSP) as adjuvants for vaccines. Vaccine 18: 238–250. 10506648 10.1016/s0264-410x(99)00195-4

[14] Garg N, Mangal S, Khambete H, Tyagi R (2010) Mucosal delivery of vaccines: role of mucoadhesive/biodegradable polymers. Recent Pat Drug Deliv Formul 4: 114–128. 20380624 10.2174/187221110791185015

[15] Borges OMF (2007) O uso de nanopartículas de quitosano, revestidas com alginato como adjuvante do antigénio da hepatite B na vacinação através das mucosas oral e nasal. Coimbra - Portugal: Universidade de Coimbra.

[16] Zaharoff DA, Rogers CJ, Hance KW, Schlom J, Greiner JW (2007) Chitosan solution enhances both humoral and cell-mediated immune responses to subcutaneous vaccination. Vaccine 25: 2085–2094. 17258843 10.1016/j.vaccine.2006.11.034 PMC1890043

[17] Reese TA, Liang H-E, Tager AM, Luster AD, Van Rooijen N, et al (2007) Chitin induces accumulation in tissue of innate immune cells associated with allergy. Nature 447: 92–96. 17450126 10.1038/nature05746 PMC2527589

[18] Yasuda K, Muto T, Kawagoe T, Matsumoto M, Sasaki Y, et al (2012) Contribution of IL-33–activated type II innate lymphoid cells to pulmonary eosinophilia in intestinal nematode-infected mice. PNAS 109: 3451–3456. 22331917 10.1073/pnas.1201042109 PMC3295287

[19] Porporatto C, Bianco ID, Correa SG (2005) Local and systemic activity of the polysaccharide chitosan at lymphoid tissues after oral administration. J Leuk Biol 78: 62–69. 10.1189/jlb.0904541 15809287

[20] Shibata Y, Honda I, Justice JP, Van Scott MR, Nakamura RM, et al (2001) Th1 Adjuvant N-Acetyl-d-Glucosamine Polymer Up-Regulates Th1 Immunity but Down-Regulates Th2 Immunity against a Mycobacterial Protein (MPB-59) in Interleukin-10-Knockout and Wild-Type Mice. Infect Immun 69: 6123–6130. 11553551 10.1128/IAI.69.10.6123-6130.2001 PMC98742

[21] Singla AK, Chawla M (2001) Chitosan: some pharmaceutical and biological aspects - an update. J Pharm Pharmacol 53: 1047–1067. 11518015 10.1211/0022357011776441

[22] Ranaldi G, Marigliano I, Vespignani I, Perozzi G, Sambuy Y (2002) The effect of chitosan and other polycations on tight junction permeability in the human intestinal Caco-2 cell line. The Journal of Nutritional Biochemistry 13: 157–167. 11893480 10.1016/s0955-2863(01)00208-x

[23] Mao H, Roy K, Troung-Le VL, Janes KA, Lin KY, et al (2001) Chitosan-DNA nanoparticles as gene carriers: synthesis, characterization and transfection efficiency. J Control Release 70: 399–421. 11182210 10.1016/s0168-3659(00)00361-8

[24] Borges O, Borchard G, Verhoef J, de Sousa A, Junginger H (2005) Preparation of coated nanoparticles for a new mucosal vaccine delivery system. Int J Pharm 299: 155–166. 15998569 10.1016/j.ijpharm.2005.04.037

[25] Borges O, Cordeiro-da-Silva A, Romeijn SG, Amidi M, Sousa AD, et al (2006) Uptake studies in rat peyer's patches, cytotoxicity and release studies of alginate coated chitosan nanoparticles for mucosal vaccination. J Control Release 114: 348–358. 16905219 10.1016/j.jconrel.2006.06.011

[26] Neutra MR, Kozlowski PA (2006) Mucosal vaccines: the promise and the challenge. Nat Rev Immunol 6: 148–158. 16491139 10.1038/nri1777

[27] Brugnerotto J, Lizardi J, Goycoolea F, Arguelles-Monal W, Desbrieres J, et al (2001) An infrared investigation in relation with chitin and chitosan characterization. Polymer 42: 3569–3580.

[28] Nakagawa Y, Murai T, Hasegawa C, Hirata M, Tsuchiya T, et al (2003) Endotoxin contamination in wound dressings made of natural biomaterials. Journal of biomedical materials research part B: Applied Biomaterials 66B: 347–355. 10.1002/jbm.b.10020 12808594

[29] USP (2005) United States Pharmacopeia,. General Chapter 85 - Bacterial Endotoxins Test

[30] Moreira C, Oliveira H, Pires LR, Simões S, Barbosa MA, et al (2009) Improving chitosan-mediated gene transfer by the introduction of intracellular buffering moieties into the chitosan backbone. Acta Biomater 5: 2995–3006. 19427930 10.1016/j.actbio.2009.04.021

[31] Oliveira C, Rezende C, Silva M, Borges O, Pêgo A, et al (2012) Oral Vaccination Based on DNA-Chitosan Nanoparticles against *Schistosoma mansoni* Infection. TSWJ 2012: 11. 10.1100/2012/938457 PMC3347803 22666171

[32] Fernandes V, Martins E, Boeloni J, Serakides R, Goes A (2012) Protective effect of rPb40 as an adjuvant for chemotherapy in experimental paracoccidioidomycosis. Mycopathologia 174: 93–105. 22391822 10.1007/s11046-012-9530-2

[33] Smithers SR, Terry RJ (1965) The infection of laboratory hosts with cercariae of *Schistosoma mansoni* and the recovery of the adult worms. Parasitology 55: 695–700. 4957633 10.1017/s0031182000086248

[34] Goes AM, Rocha RS, Gazzinelli G, Doughty BL (1989) Production and characterization of human monoclonal antibodies against Schistosoma mansoni. Parasite Immunology 11: 695–711. 2694076 10.1111/j.1365-3024.1989.tb00930.x

[35] Tang ESK, Huang M, Lim LY (2003) Ultrasonication of chitosan and chitosan nanoparticles. Int J Pharm 265: 103–114. 14522123 10.1016/s0378-5173(03)00408-3

[36] Bergquist R, Utzinger J, McManus DP (2008) Trick or Treat: The Role of Vaccines in Integrated Schistosomiasis Control. PLoS Negl Trop Dis 2: e244. 18575619 10.1371/journal.pntd.0000244 PMC2430529

[37] Kotton CN, Hohmann EL (2004) Enteric Pathogens as Vaccine Vectors for Foreign Antigen Delivery. Infect Immun 72: 5535–5547. 15385450 10.1128/IAI.72.10.5535-5547.2004 PMC517600

[38] Benesch J, Tengvall P (2002) Blood protein adsorption onto chitosan. Biomaterials 23: 2561–2568. 12033604 10.1016/s0142-9612(01)00391-x

[39] Li XY, Kong X, Shi S, Zheng X, Guo G, et al (2008) Preparation of alginate coated chitosan microparticles for vaccine delivery. BMC Biotechnol 8: 89. 19019229 10.1186/1472-6750-8-89 PMC2603011

[40] O'Hagan DT, Rappuoli R (2004) Novel approaches to vaccine delivery. Pharm Res 21: 1519–1530. 15497674 10.1023/B:PHAM.0000041443.17935.33 PMC7088827

[41] Wang J, Zeng Z, Xiao R, Xie T, Zhou G, et al (2011) Recent advances of chitosan nanoparticles as drug carriers. Int J Nanomedicine 6: 765–774. 21589644 10.2147/IJN.S17296 PMC3090273

[42] Chopra S, Mahdi S, Kaur J, Iqbal Z, Talegaonkar S, et al (2006) Advances and potential applications of chitosan derivatives as mucoadhesive biomaterials in modern drug delivery. Journal of Pharmacy and Pharmacology 58: 1021–1032. 16872548 10.1211/jpp.58.8.0002

[43] Prego C, Torres D, Alonso MJ (2005) The potential of chitosan for the oral administration of peptides. Expert Opinion on Drug Delivery 2: 843–854. 16296782 10.1517/17425247.2.5.843

[44] Cui Z, Mumper R (2001) Chitosan-based nanoparticles for topical genetic immunization. J Control Release 75: 409–419. 11489327 10.1016/s0168-3659(01)00407-2

[45] Lubben M, Kersten G, Fretz M, Beuvery C, Verhoef J, et al (2003) Chitosan microparticles for mucosal vaccination against diphtheria: oral and nasal efficacy studies in mice. Vaccine 21: 1400–1408. 12615436 10.1016/s0264-410x(02)00686-2

[46] Periwal SB, Speaker TJ, Cebra JJ (1997) Orally administered microencapsulated reovirus can bypass suckled, neutralizing maternal antibody that inhibits active immunization of neonates. J Virology 71: 2844–2850. 9060640 10.1128/jvi.71.4.2844-2850.1997 PMC191409

[47] Bowersock TL, HogenEsch H, Suckow M, Porter RE, Jackson R, et al (1996) Oral vaccination with alginate microsphere systems. J Control Release 39: 209–220.

[48] Fujihara M, Nagumo T (1993) An influence of the structure of alginate on the chemotactic activity of macrophages and the antitumor activity. Carbohydrate Research 243: 211–216. 8324764 10.1016/0008-6215(93)84094-m

[49] Mata E, Igartua M, Patarroyo M, Pedraz J, Hernández R (2011) Enhancing immunogenicity to PLGA microparticulate systems by incorporation of alginate and RGD-modified alginate. Eur J Pharm Sci 44: 32–40. 21699977 10.1016/j.ejps.2011.05.015

[50] Wynn TA, Cheever AW (1995) Cytokine regulation of granuloma formation in schistosomiasis. Curr Opin Immunol 7: 505–511. 7495514 10.1016/0952-7915(95)80095-6

[51] Jarnicki AG, Conroy H, Brereton C, Donnelly G, Toomey D, et al (2008) Attenuating Regulatory T Cell Induction by TLR Agonists through Inhibition of p38 MAPK Signaling in Dendritic Cells Enhances Their Efficacy as Vaccine Adjuvants and Cancer Immunotherapeutics. The Journal of Immunology 180: 3797–3806. 18322186 10.4049/jimmunol.180.6.3797

[52] Slutter B, Jiskoot W (2010) Dual role of CpG as immune modulator and physical crosslinker in ovalbumin loaded N-trimethyl chitosan (TMC) nanoparticles for nasal vaccination. J Control Release 148: 117–121. 20600405 10.1016/j.jconrel.2010.06.009

[53] Muzzarelli R (2010) Chitins and Chitosans as Immunoadjuvants and Non-Allergenic Drug Carriers. Mar Drugs 8: 292–312. 20390107 10.3390/md8020292 PMC2852840

[54] Wilson MS, Mentink-Kane MM, Pesce JT, Ramalingam TR, Thompson R, et al (2007) Immunopathology of schistosomiasis. Immunol Cell Biol 85: 148–154. 17160074 10.1038/sj.icb.7100014 PMC3437548

[55] Wynn TA (1996) Development of an Antipathology Vaccine for Schistosomiasis. Annals N Y Acad Sci 797: 191–195. 10.1111/j.1749-6632.1996.tb52960.x 8993362

[56] Gordon S (2003) Alternative activation of macrophages. Nat Rev Immunol 3: 23–35. 12511873 10.1038/nri978

[57] Voehringer D, Reese TA, Huang X, Shinkai K, Locksley RM (2006) Type 2 immunity is controlled by IL-4/IL-13 expression in hematopoietic non-eosinophil cells of the innate immune system. The Journal of Experimental Medicine 203: 1435–1446. 16702603 10.1084/jem.20052448 PMC2118302

[58] Da Silva CA, Hartl D, Liu W, Lee CG, Elias JA (2008) TLR-2 and IL-17A in Chitin-Induced Macrophage Activation and Acute Inflammation. J Immunol 181: 4279–4286. 18768886 10.4049/jimmunol.181.6.4279 PMC2577310

[59] Herbert DBR, Hölscher C, Mohrs M, Arendse B, Schwegmann A, et al (2004) Alternative Macrophage Activation Is Essential for Survival during Schistosomiasis and Downmodulates T Helper 1 Responses and Immunopathology. Immunity 20: 623–635. 15142530 10.1016/s1074-7613(04)00107-4

[60] Wilson RA, Coulson PS (1998) Why Don't We Have a Schistosomiasis Vaccine? Parasitology Today 14: 97–99. 17040714 10.1016/s0169-4758(97)01198-8

